# *Lactobacillus plantarum* PMO 08 as a Probiotic Starter Culture for Plant-Based Fermented Beverages

**DOI:** 10.3390/molecules25215056

**Published:** 2020-10-30

**Authors:** Young Joo Oh, Tae Seok Kim, Hwang Woo Moon, So Young Lee, Sang Yun Lee, Geun Eog Ji, Keum Taek Hwang

**Affiliations:** 1 Pulmuone Co., Ltd., Cheongju 28164, Korea; tskim@pulmuone.com (T.S.K.); peter.moon@pulmuone.com (H.W.M.); molly.lee@pulmuone.com (S.Y.L.); sylee@pulmuone.com (S.Y.L.); 2 Department of Food and Nutrition, and Research Institute of Human Ecology, Seoul National University, Seoul 08826, Korea; geji@snu.ac.kr

**Keywords:** non-dairy probiotic beverage, *L. plantarum* PMO 08, probiotic starter culture, kimchi, plant-based fermented beverage, tomato juice

## Abstract

*Lactobacillus plantarum* PMO 08 was evaluated as a starter culture for plant-based probiotic beverages. Its viability under various culture conditions and acidification ability in standardized tomato medium, fermentation parameters, and beverage properties were assessed. *Lactobacillus plantarum* PMO 08 could grow under various culture conditions; there was a high correlation between the incubation time to reach the optimal conditions and the inoculation concentration of lactic acid bacteria (LAB) (r^2^ = 0.997). Acidity (0.958 ± 0.002%) and LAB count (9.78 ± 0.14 Log_10_ CFU/mL) were significantly higher when fermented with *L. plantarum* than with the yogurt starter culture. A survival rate of 96% and 95% in artificial gastric juice and artificial intestinal juice, respectively, indicated that the probiotic requirements were met. The total polyphenol and glutamine content, and antioxidant activity increased after fermentation. The proline content significantly increased in *L. plantarum* PMO 08- fermented beverage. Thus, *L. plantarum* PMO 08 is an effective starter culture for non-dairy probiotic beverages whose functional quality may be improved by fermentation.

## 1. Introduction

Significant growth in the global probiotics market has increased the demand for plant-based probiotic drinks as a replacement for conventional dairy products [1]. The word “probiotics” is defined as “live microorganisms conferring a health benefit on the host when administered in adequate amounts” [2]. Probiotics are generally provided in the form of supplements (powders, capsules, and tablets) but have gradually diversified to cater to consumer preferences for convenient intake and enhanced functional quality; for example, fermented dairy products with added probiotic cultures, probiotic shots ingested as a liquid containing lactic acid bacteria (LAB), and various liquid carriers of probiotic products such as probiotic beverages mixed with LAB-fermented oats [3,4].

Compared with fermented milk products, plant-based probiotic drinks do not contain dairy ingredients, thereby eliminating concerns related to lactose intolerance and animal cholesterol and fat [5]. Other health benefits of plant-based fermented products include the supply of micronutrients such as antioxidant vitamins, bioactive compounds, and minerals, as well as an increased absorption rate and physiological activity of flavonoid components through LAB fermentation, which improves intestinal health and enhances antioxidant activity and immune functions [6,7]. As the polyphenols contained in plants act as prebiotics to enhance the growth of beneficial human gut bacteria and their adhesion to enterocytes, it is expected that plant-based probiotic beverages can also be offered as synbiotics [8]. Early research focused on the substrates used to produce plant-based probiotic beverages as substitutes for fermented milk, such as plant protein and carbohydrate sources such as soybean, oat, and barley [9]. However, recent studies have attempted to expand plant materials to include fruits, vegetables, and teas rich in polyphenols, such as mulberry, broccoli, and green tea, respectively [10,11,12].

To acquire microorganisms suitable for plant fermentation, it is necessary to isolate LAB from a various plant sources, including traditional fermented foods, and determine their probiotic characteristics [9]. Despite such efforts, only limited industrialized probiotic starter cultures are currently available. *Lactobacillus plantarum* isolated from plants is more suitable for plant fermentation because it can better adapt to matrixes with hostile conditions for microorganisms, such as those related to organic acids, polyphenols, and the pH of fruits and vegetables [13]. *Lactobacillus plantarum* PMO 08 is a type of LAB isolated from kimchi, a traditional Korean food, and characterized by excellent bile acid deconjugation ability [14]. According to a preclinical study using a high fat diet (45% fat diet), *L. plantarum* PMO 08 exhibits anti-cholesterol effect by regulating the expression of SREBP-2 mRNA, a transcription factor involved in cholesterol metabolism [15]. In an artificial gastrointestinal juice, *L. plantarum* PMO 08 showed a higher survival rate than *L. casei* isolated from dairy products, as well as excellent adhesion to enterocytes, thereby meeting the probiotic requirements [16]. Furthermore, Jang et al. [17] reported that *L. plantarum* PMO 08 suppressed compound 48/80 or histamine-induced pruritus in a dose-dependent manner, and this was ascribed to its ability to regulate anti-inflammatory cytokines such as TNF-a and IL-4, thus suppressing hypersensitivity. Therefore, given the excellent anti-cholesterol and immune hypersensitivity suppression effects of *L. plantarum* PMO 08, its feasibility as a starter culture and its ability to provide probiotic beverages with live bacteria by fermenting plant materials were assessed in the present study.

Tomato is commonly used as the substrate to study plant fermentation quality because it is easy to handle, available worldwide, and appropriate for LAB fermentation [18,19]. It also has health benefits as a superfood rich in nutrients such as lycopene, naringenin, and vitamin C [18]. As it contributes to the prevention of lifestyle diseases such as obesity and cardiovascular disease, tomato is an excellent carrier for probiotics [20].

In the present study, we aimed to develop a plant-based probiotic beverage using *L. plantarum* PMO 08 isolated from kimchi, a traditional Korean fermented food. To investigate its usability as a starter culture, we verified the growth characteristics and acidification ability of *L. plantarum* PMO 08 and measured the titratable acidity (TA), viable LAB count, and free amino acid content in fermented tomato juice, all used as indicators of fermentation quality. We then compared the antioxidant activity, total polyphenol content, and gastrointestinal tolerance of the fermented tomato juice produced using *L. plantarum* PMO 08 with that produced using yogurt starter culture.

## 2. Results

### 2.1. Effect of Incubation Temperature, pH, and NaCl Concentration on L. plantarum PMO 08 Viability

**Figure 1:**
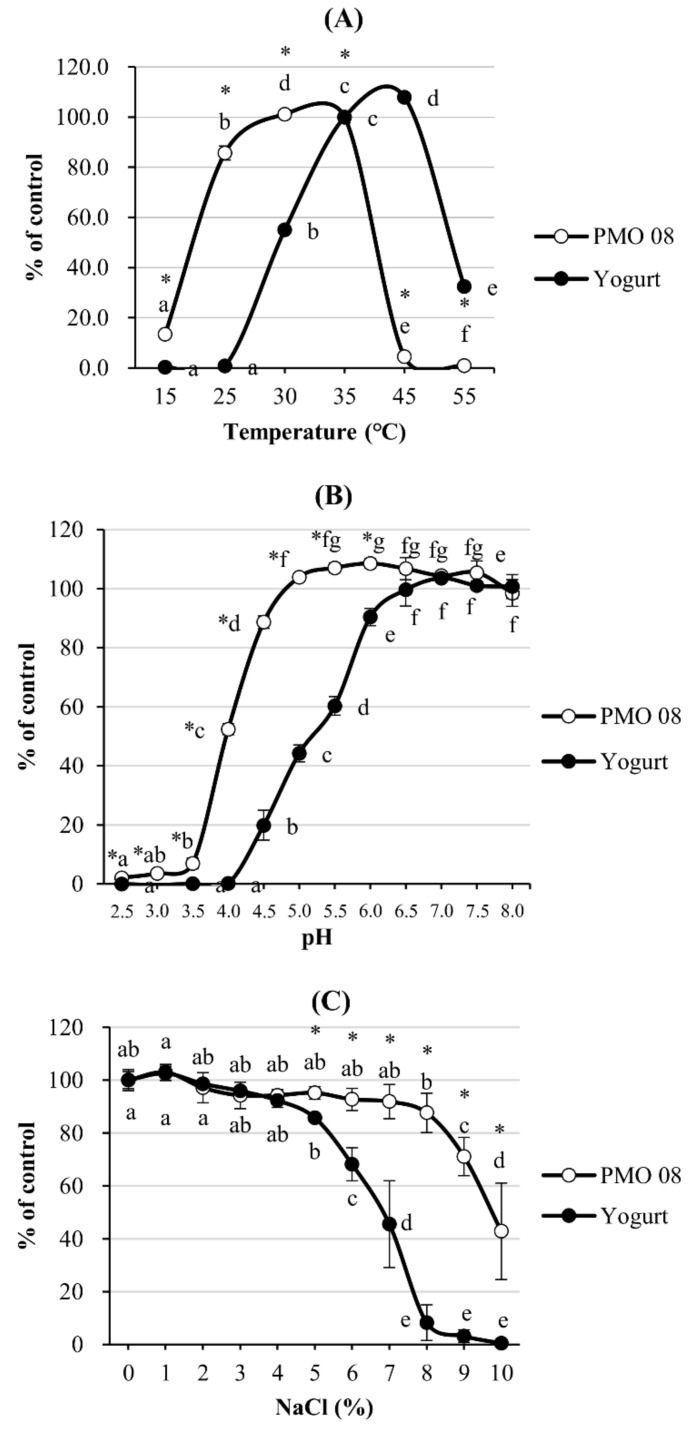
Effect of culture conditions on bacterial cell growth. (**A**) Temperature, (**B**) pH, and (**C**) NaCl concentration. PMO 08: *Lactobacillus plantarum* PMO 08; Yogurt: Starter culture mixture of *Lactobacillus acidophilus, Streptococcus thermophilus,* and *Bifidobacterium lactis*. All values are presented as mean ± standard deviation (n = 3). Values with different letters are significantly different at *p* < 0.05 according to Duncan’s multiple range test. * Significant difference between PMO 08 and yogurt (*p* < 0.05).

Figure 1A shows the effect of incubation temperature on LAB viability, which was used to identify the optimal incubation temperature of *L. plantarum* PMO 08. Cell growth peaked at 30 °C (102.2 ± 1.7%), and it was suppressed to 4.4% at 45 °C. In contrast, yogurt starter showed a higher growth rate [107.9 ± 1% (*p* < 0.05)] at 45 °C than at 37 °C, and cell growth was suppressed at 25 °C or below. The acid resistance of *L. plantarum* PMO 08 culture was estimated by measuring its viability for 24 h in MRS broth while varying the pH from 2.5 to 8.0, as shown in Figure 1B. The optimal pH of *L. plantarum* PMO 08 was between 5.0 and 7.5, whereas that of yogurt starter culture was between 6.5 and 8.0. The salt tolerance of *L. plantarum* PMO 08 was examined by measuring LAB viability in MRS broth while varying the NaCl concentration from 1 to 10%, as shown in Figure 1C. *Lactobacillus plantarum* PMO 08 showed the highest LAB viability in MRS broth with 1% NaCl (103.2%), and no significant growth inhibition was observed in broth with NaCl up to a concentration of 8%. For the yogurt starter culture, the maximum growth rate was observed at 1% NaCl, similar to *L. plantarum* PMO 08, but significant growth suppression was observed above 5% NaCl concentration. These results indicated that *L. plantarum* PMO 08 can be used as a starter culture in a wide range of plant-substrate environments, given its viability across broad ranges of pH and salt concentrations.

### 2.2. Acidification Ability of Plant-Based Substrates

**Figure 2:**
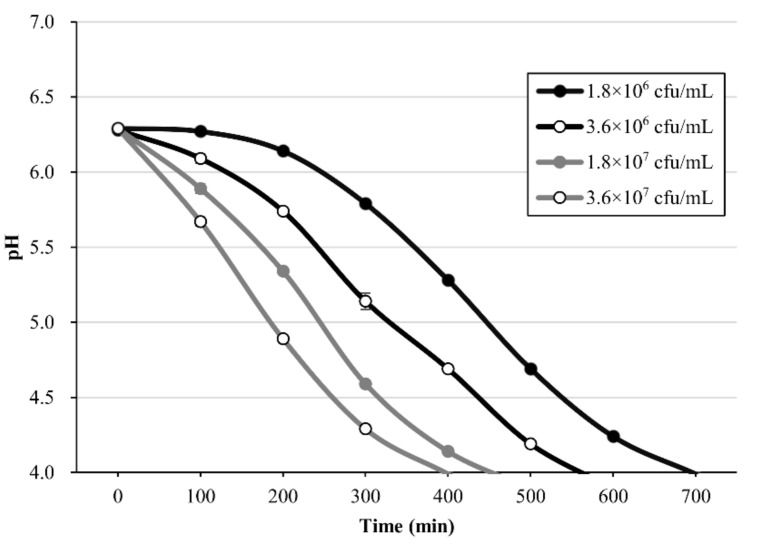
Acidification curves for different inoculation levels of *L. plantarum* PMO 08 in diluted tomato juice. All values are presented as mean ± standard deviation (n = 3). Fermentation was performed at 37 °C.

A standardized plant-based substrate was prepared to use *L. plantarum* PMO 08 as a starter culture. Figure 2 presents the measurement of acidification ability. By monitoring the time required for the tomato juice to reach pH 4.0 after LAB inoculation, we confirmed that the acidification ability increased with the increase the LAB inoculation amount. A very high correlation (r^2^ = 0.997) was observed between the incubation time required to reach pH 4 and the LAB inoculation concentration, thus verifying the feasibility of *L. plantarum* PMO 08 as a starter culture.

### 2.3. Physicochemical and Microbial Characterization of the Plant-Based Probiotic Beverages

Table 1 presents the results of quality comparison of the fermented probiotic beverages containing live bacteria manufactured for testing. The plant-based probiotic beverage prepared using *L. plantarum* PMO 08 presented a solid content of 4.63 ± 0.03 °Brix (%), acidity of 0.958 ± 0.002%, a pH of 3.39 ± 0.03, and a LAB count of 9.78 ± 0.14 Log_10_ CFU/mL; the LAB count and acidity were significantly higher than those of the beverage prepared using the yogurt starter culture (*p* < 0.05). It was confirmed that both non-fermented and fermented beverages were not contaminated by total aerobic bacteria, *Escherichia coli*/coliforms, yeast, and molds.

### 2.4. Viability in Artificial Gastrointestinal Juice

**Figure 3:**
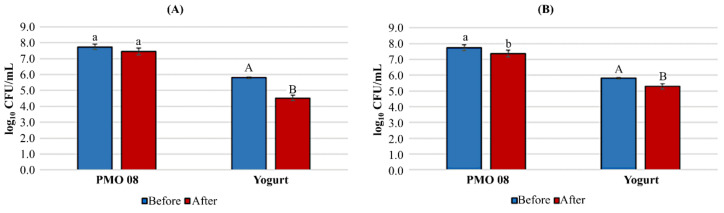
Viability of fermented beverages in the artificial gastrointestinal juice. (**A**) Artificial gastric juice condition (pH 2.5) with 0.3% pepsin. (**B**) 0.45% bile salts condition with 0.1% pancreatin. PMO 08: Fermented beverage with *L. plantarum* PMO 08; Yogurt: Fermented beverage with yogurt starter culture containing *Lactobacillus acidophilus, Streptococcus thermophilus,* and *Bifidobacterium lactis*. The viable count of lactic acid bacteria was determined by plating the cells from each stage on MRS agar followed by incubation at 37 °C for 48 h. Before: Viable cell count of each fermented beverage before the test; after: Viable cell count of each fermented beverage after the test. Values are represented as the mean of three replicates and error bars represent the standard deviation. Different lowercase and uppercase letters on the error bars indicate significant differences in PMO 08 and yogurt starter culture, respectively (*p* < 0.05).

The viability of the plant-based probiotic beverages in the artificial gastrointestinal juice is presented in Figure 3. The viability of the probiotic beverage fermented with *L. plantarum* PMO 08 in artificial gastric juice and artificial intestinal juice was up to 96.2 ± 2.3% and 95.3 ± 2.51%, respectively. The survival rate of the probiotic beverage fermented with the yogurt starter culture was 78.0 ± 3.5% in artificial gastric juice, which was significantly lower (*p* < 0.05) than that of the beverage fermented with *L. plantarum* PMO 08, at 91.0 ± 3.6%, in artificial intestinal juice, and this was similar to that of the beverage fermented with *L. plantarum* PMO 08.

### 2.5. Polyphenol Content and Antioxidant Activity

**Figure 4:**
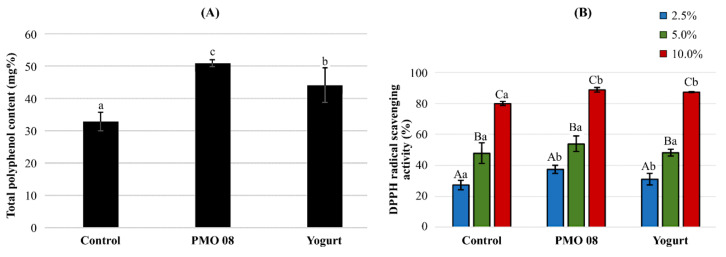
Changes in the total polyphenol content and antioxidant activity during fermentation. (**A**) Total polyphenol content. Values are represented as the mean of three replicates and error bars represent the standard deviation. (**B**) 1,1-Diphenyl-2-picrylhydrazyl radical scavenging activity. Control: Non-fermented tomato juice beverage; PMO 08: Tomato juice fermented by *L. plantarum* PMO 08; Yogurt: Tomato juice fermented by yogurt starter culture containing *Lactobacillus acidophilus, Streptococcus thermophilus*, and *Bifidobacterium lactis*. 1st column (blue), 2.5% of each beverage concentration (v/v); 2nd column (green), 5% of each beverage concentration (v/v); 3rd column (red), 10% of each beverage concentration (v/v). Each of the beverages was diluted with purified water. Different uppercase letters denote significant differences between different concentrations of the same type of fermented beverage. Different lowercase letters denote significant differences between the three types of fermented beverages (*p* < 0.05).

The polyphenol content of the plant-based probiotic beverage increased by 154.7% when fermented with *L. plantarum* PMO 08 and 134.1% when fermented with the yogurt starter culture (Figure 4A). The results of the DPPH radical scavenging capacity assay are illustrated in Figure 4B. Test samples were prepared by diluting each beverage to 10%, 5%, and 2.5% (*v/v*) with purified water, and then, the DPPH radical scavenging activity was evaluated at different concentrations. The DPPH scavenging activity significantly increased in a dose-dependent manner (*p* < 0.05). DPPH free radical scavenging ranged from 37.29% to 88.92% and from 31.02% to 87.38% in beverages fermented with *L. plantarum* PMO 08 and beverages fermented with yogurt starter culture, respectively.

### 2.6. Free Amino Acid Composition

Table 2 outlines the free amino acid content in the plant-based probiotic beverages. In both non-fermented and fermented tomato beverages, glutamic acid was the major amino acid, followed by aspartic acid and alanine. After fermentation, all amino acid content decreased, except glutamine and proline. In particular, in tomato beverages fermented with *L. plantarum* PMO 08, the content of isoleucine, valine, and methionine decreased by 100%. Conversely, the glutamine content in the plant-based probiotic beverages increased by 405% (*p* < 0.05) when fermented with *L. plantarum* PMO 08 and 1849% (*p* < 0.05) when fermented with the yogurt starter culture. Furthermore, there was a significant increase in proline content in *L. plantarum* PMO 08- fermented beverage (*p* < 0.05).

## 3. Discussion

In all probiotic products including fermented milk, viable LAB is a key quality indicator; thus, it is crucial to increase the count of viable LAB in the final product [3,21]. In the present study, *L. plantarum* PMO 08 presented the highest LAB count at 30 °C, and cell growth occurred at pH 3.5–8.0 and with 0–8.0% NaCl. Thus, the characteristics of PMO 08 are suitable for the fermentation of vegetables and fruits with a wide range of pH and inorganic salt concentrations. At the end of fermentation, *L. plantarum* PMO 08 culture presented a pH of 3.39 and TA of 0.958%, releasing lactic acid, which imparts a sour taste and an unfavorable environment for spoilage bacteria [22]. A rapid decrease in pH is particularly important for the probiotic functions, whereby foods in a pH range of 3.5–4.5 lower than the pH in the gastrointestinal juice, suppress cell growth of harmful bacteria, and improve probiotic effects by enhancing the survival rate of beneficial bacteria [23].

In the present study, plant-based probiotic beverages were produced using tomato as a culture substrate. *Lactobacillus plantarum* PMO 08 satisfied the international requirements for fermented milk with a LAB count of 9.78 Log_10_ CFU/mL. A high viable LAB count is essential for acidification, which substantially affects the sensory quality of fermented beverages [10]. A previous study on LAB fermentation using tomato juice reported a relatively high LAB count of 8.0–9.9 Log_10_ CFU/mL, pH of 3.2–4.0, and TA of ≥0.65%, exhibiting good agreement with the results of the present study [19]. Owing to its high viable LAB count tomato juice fermented with LAB, is a suitable replacement to fermented milk for vegetarians or consumers with lactose intolerance [24].

For probiotics to be effective, they should not only contain a high number of viable bacterial cells, but also maintain a high gastrointestinal survival rate. Therefore, the survival rate of LAB in the artificial gastrointestinal juice is an important indicator affecting the potential probiotic performance, and a starter culture must withstand the physicochemical barriers of the gastrointestinal tract such as acid, digestive enzymes (e.g., pepsin), and bile acid [25,26]. Similar to the results in the artificial gastrointestinal juice [16], the probiotic beverage fermented by *L. plantarum* PMO 08 exhibited a high survival rate (96%) in the artificial gastric juice and it was resistant to 0.45% bile salts. Therefore, tomato culture can be used as a carrier for probiotics, and *L. plantarum* PMO 08 is an effective a probiotic starter culture. Conversely, the yogurt LAB presented a survival rate in artificial gastric juice of 78%, indicating the limitations of some starter culture LAB such as *Streptococcus thermophilus* [27].

Amino acid metabolism induced by fermentation is a major factor affecting food taste, nutrition, and safety [28]. In the present study, the total free amino acid content decreased during tomato fermentation with *L. plantarum* PMO 08, suggesting that free amino acids were used as a nitrogen source. In contrast, the proline and glutamine content was significantly increased in the beverage fermented by *L. plantarum* PMO 08. Proline, a well-known key amino acid in bacteria, fungi, and plants, functions as a stress protectant by preserving the membrane integrity and preventing protein aggregation [29,30]. Additionally, the exogenous addition of proline under hyperosmotic condition could improve cell growth, lactic acid production, and preservation [31]. Under stress conditions such as glucose starvation, *L. plantarum* B21 upregulated the proline content in culture media [32]. Therefore, proline is considered to contribute to the adaptability of *L. plantarum* PMO 08 under stress, leading to a higher viable cell count and survival rate in the gastrointestinal tract than the yogurt starter culture. Glutamine, which is an amino acid synthesized by glutamate synthase from glutamic acid and ammonia (NH_3_), is involved in nitrogen exchange via NH_3_ and pH homeostasis in organisms, and it serves as the nucleotide substrate of purines, pyrimidines, and amino sugars in most cells [33]. Glutamine is an important fuel for small intestinal cells and immune cells, and it plays an important role in intestinal surface integrity [34]. More specifically, as an immunomodulatory nutrient, glutamine regulate tight junction proteins (occludin, claudin-1, and zona occludens-1), controls chain reactions of cytokines such as TNF-α and IL-17A, and increases local secretory IgA (sIgA) production for preventing infection [35]. A previous study using *L. plantarum* PMO 08 reported an improvement in pruritus by regulating of pro-inflammatory cytokines such as IL-4 and TNF-a [17]. Therefore, further research is required to investigate the mechanism of action of *L. plantarum* PMO 08 by comparing intestinal glutamine concentration and its immunomodulatory effects in the body.

The functional properties of probiotics can vary depending on the carrier foods or accompanying ingredients [36]. Tomato is rich in antioxidants such as lycopene, beta carotene, vitamin C, and polyphenols, and it can be used to produce fermented beverages with functional ingredients enhanced by LAB fermentation [37,38]. Moreover, some of the polyphenol components of plants act as prebiotics, thereby increase the cell growth rate and adhesion of beneficial bacteria to enterocytes, with the potential to act as synbiotics [8]. In the present study, tomato fermentation with *L. plantarum* PMO 08 resulted in a 154.7% increase in the total polyphenol content and a concentration-dependent increase in the DPPH radical-scavenging activity compared with pre-fermentation levels. This aspect is consistent with the mobilization of polyphenols to enhance antioxidant activity during fermentation [39], which can be explained by the increased antioxidant ability resulting from the polymerization of polyphenol compounds [40]. Furthermore, Ricci et al. [41] reported that the fermentation of cherry juice with *L. plantarum* strains isolated from different sources did not change the total polyphenol content, but it altered polyphenol metabolite distribution in each LAB species, revealing that polyphenol availability varies with *L. plantarum* strain [41]. Specifically, while *L. plantarum* commonly produces p-hydroxyphenyllactic acid and phenyllactic acid, other acids such as *p*-coumaric acid, caffeic acid, and dihydrocaffeic acid vary in their availability according to *L. plantarum* strain. Therefore, follow-up studies are necessary to determine the key functional indicators of polyphenol metabolites and DPPH radical- scavenging activity during tomato fermentation with *L. plantarum* PMO 08.

A limitation of the present study is that the yogurt strain did not allow easy comparison of the fermentation quality as it contained different species to PMO 08; however, the results confirmed the need to develop strains suitable for plant-based starter cultures.

In addition, compared with the commercialized yogurt starter culture, the PMO 08 probiotic beverage showed a significantly higher TA and viable cell count, but a similar antioxidant activity. Therefore, future research on the optimal culture conditions also is necessary to identify the substances contributing to the antioxidant activity and help increase their content Moreover, it was not determined whether the plant-based probiotic beverages produced with this starter culture have more health benefits; thus, in vivo studies on the bioactivity suitable for the target function are required.

## 4. Materials and Methods

### 4.1. Bacterial Strains

*Lactobacillus plantarum* PMO 08 was obtained from Pulmuone Co., Ltd. (Cheongju, Republic of Korea) and the yogurt starter culture (*S. thermophilus*, *L. acidophilus,* and *Bifidobacterium lactis* BB-12) was obtained from Chr. Hansen Holding A/S (Hoersholm, Denmark). Before use, each LAB culture was incubated at 37 °C for 24 h in Man Rogosa Sharpe (MRS) broth (Difco Laboratories Inc., Detroit, MI, USA).

### 4.2. Effect of Temperature, pH, and NaCl Concentration on Bacterial Viability

The effects of incubation temperature was determined in the range of 15 to 55 °C, and those of pH was determined in the range 2.5–8.0 using 1 N NaOH and 1 N HCl. The salt concentration-dependent viability was measured using the MRS broth with NaCl at concentrations of 1.0–10.0%. The viability of *L. plantarum* PMO 08 and yogurt starter culture strains was compared by measuring the absorbance of the sample at 600 nm using a UV-VIS spectrophotometer (VersaMax, Molecular Devices, CA, USA).

### 4.3. Preparation of Plant Culture Substrate and Acidification Ability

To test the activity of *L. plantarum* PMO 08 as a starter culture, a standardized plant-based substrate was prepared, and its acidification ability was evaluated. As plant-based substrate, tomato was considered suitable for LAB fermentation based on previous research and on its global availability worldwide [18]. A standardized tomato substrate was used after dilution to 5 °Brix by adding purified water to commercial tomato paste (Empresas Carozzi S.A., Antofagasta, Chile), the pH of the solution was set to 6.5 ± 0.5 with 1 N NaOH, Thereafter, the solution was sterilized at 121 °C for 15 min. The acidification ability of *L. plantarum* PMO 08 was evaluated by measuring the time required to reach a pH of 4.0 at different inoculation amounts of *L. plantarum* PMO 08.

### 4.4. Preparation of a Plant-Based Probiotic Beverage

The prepared tomato paste was diluted to reach 5 °Brix by adding purified water, passed through a homogenizer (BBX24B-CE; Next Advance, NY, USA) under a pressure of 200 ± 20 bar and sterilized for 30 min at 105 °C. For inoculation, *L. plantarum* PMO 08 and yogurt starter LAB were diluted to a concentration of 10^7^ CFU/mL, inoculated, and then cultured at 37 °C for 24 h in a constant temperature chamber. To compare the physicochemical and microbial properties before and after fermentation, tomato paste beverage without LAB inoculation was used as the control.

### 4.5. Analysis of Brix, pH, and Titratable Acidity

The properties of the fermented beverages produced were investigated by measuring the sugar content using a refractometer (RX-5000; Atago, Tokyo, Japan); the mean of three replicates was expressed as °Brix (%). The pH of the sample was measured using a pH meter (Orion 2 STAR; Thermo Fisher Scientific, Waltham, MA, USA). Acidity was measured using the titration method. Briefly, 1 mL of the sample was diluted with 49 mL of distilled water and titrated to pH 8.3 using 0.1 N NaOH solution. Acidity is expressed as a percentage of lactic acid. The amount of 0.1 N NaOH used for titration was calculated and converted to acidity (in percentage) using the following formula [1]:

(1) $$\mathrm{acidity}\;\left(\%\right)\;=\;\left[\left(\mathrm{mL}\;\mathrm{of}\;0.1\;N\;\mathrm{NaOH}\;\times \;\mathrm{normality}\;\mathrm{of}\;\mathrm{NaOH}\;\times \;0.009\right)\;/\;\mathrm{weight}\;\mathrm{of}\;\mathrm{sample}\;\left(g\right)\right]\;\times \;100$$

### 4.6. Microbial Analysis

*Escherichia coli*/coliforms, yeast and molds, and LAB in the probiotic beverages were enumerated. Each beverage was serially diluted with sterilized saline. Subsequently, 1 mL of each diluted sample was added onto 3 M Petrifilm™ plates (3 M Corp., St. Paul, MN, USA) to enumerate *E. coli*/coliforms and yeast and molds, and inoculated on MRS agar plates (Difco Laboratories Inc., Detroit, MI, USA) to enumerate LAB. Enumeration of *E. coli*/coliforms and LAB was performed after incubation at 37 °C for 48 h, whereas that of yeast and molds was carried out after incubation at 25 °C for 3–5 days. In the non-fermented tomato juice (control group), the total bacterial count was determined instead of LAB count to verify the sterilization effect, and other microbial analyses were the same as those of the fermented juice. The total bacterial counts were determined using 3 M Petrifilm™ Aerobic Count plate, and the enumeration of total bacteria was performed after incubation at 37 °C for 48 h. The results are expressed as log colony-forming units (CFU) per milliliter of juice.

### 4.7. Tolerance to Artificial Gastrointestinal Juice

Tolerance to artificial gastrointestinal fluid was determined as described by Valero-Cases et al. [35]. Artificial gastric juice and artificial intestinal fluid were tested separately. Briefly, the tolerance of the beverages to artificial gastric juice was determined at a controlled pH of 2.5 by incubating the beverages in PBS containing 0.3% (*w/v*) pepsin (Sigma-Aldrich, St. Louis, MO, USA) at 37 °C for 2 h. The tolerance to artificial intestinal juice was determined after exposure to PBS consisting of 0.45% (*w/v*) bile salts (Sigma-Aldrich, St. Louis, MO, USA) and 0.1% pancreatin (Sigma-Aldrich), adjusted to pH 7.0 for 2 h at 37 °C. The number of viable cells was determined by incubating aliquots on MRS agar plates at 37 °C for 48 h. The viable bacterial count after each stress treatment is expressed in Log_10_ CFU. The survival rate (%) was determined by comparing the Log_10_ number of viable bacterial cells after incubation (N) to the Log_10_ initial number of viable bacterial cells (N0):

(2) $$\mathrm{Survival}\;\mathrm{rate}\;\left(\%\right)\;=\;\mathrm{Log}\;N/\mathrm{Log}\;N0\;\times \;100$$

### 4.8. DPPH Radical Scavenging Activity

The DPPH radical scavenging activity was assessed using the method of Blois et al. [42]. In brief, 2.0 mL of the beverage at different concentrations (10%, 5%, and 2.5% (*v/v*)) in purified water was mixed with an equal volume of freshly prepared 0.469 mM DPPH in ethanol (Samchun Chemical Co., Ltd., Pyeongtaek, South Korea). After 30 min of incubation in dark at 25 °C, the reaction mixture was centrifuged at 8000× *g* for 10 min at 4 °C. The absorbance of the upper supernatant was measured at 517 nm. The DPPH radical scavenging activity (%) of samples was calculated using the following formula:

(3) $$\mathrm{DPPH}\;\mathrm{radical}\;\mathrm{scavenging}\;\mathrm{effect}\;\left(\%\right)\;=\;\left[1-\left(\mathrm{OD}\;\mathrm{Sample}\;/\mathrm{OD}\;\mathrm{Control}\right)\right]\;\times \;100$$

where OD sample is the optical density of the sample mixed with DPPH solution and OD control is the optical density of the distilled water with DPPH solution.

### 4.9. Measurement of Total Polyphenol Content

To measure the total polyphenol content, a solution was prepared by dissolving each sample (0.01 mL) in purified water. To the solution, mL of Folin–Ciocalteu reagent was added, followed by 20% sodium carbonate (0.15 mL). The mixture was allowed to react at room temperature for 40 min. The absorbance was then measured at 725 nm. Gallic acid (Sigma Chemical Co., St. Louis, MO, USA) was used as a standard.

### 4.10. Measurement of Free Amino Acid Composition

Amino acid composition was determined as previously reported [43] using HPLC 1260 (Agilent Technologies, CA, USA) equipped with the Capcell Pak C18 UG 120 column (4.6 mm internal diameter, 150 mm length, and 5 μm particle size). An analytical detector was used to measure all amino acids at 338 nm, except proline (262 nm). The concentration of amino acids is expressed as mg/100 g sample.

### 4.11. Statistical Analyses

SPSS 22.0 (IBM Corp., Armonk, NY, USA) was used for all statistical analyses. For all experiments, a one-way analysis of variance followed by Duncan’s multiple range test or Student’s *t*-test were used to assess statistical significance. Data are expressed as mean and standard deviation of three replications; differences were considered statistically significant at *p* < 0.05.

## 5. Conclusions

The results of the present study confirmed that *L. plantarum* PMO 08 can be grown under a wide range of culture conditions: pH 3.5–8.0, incubation temperature 15–35 °C, and salt concentration 0–8%. A high LAB content of 9 Log_10_ CFU/mL or more, and LAB stability at low pH indicated the feasibility of using *L. plantarum* PMO 08 as a starter culture. Furthermore, a high survival rate in the artificial gastrointestinal juice was confirmed, satisfying the basic requirements for probiotics. In addition to the fermentation-induced supply of viable LAB cells through an increase in the total polyphenol content, antioxidant activity, and glutamine content, *L. plantarum* PMO 08 was found to be useful for health promotion. To ensure the future application of *L. plantarum* PMO 08 as a probiotic starter culture in the non-dairy probiotics market, future research should determine its fermentation quality conformance using various plant sources and optimize the fermentation conditions for bioactive components or bioactivity.

## Figures and Tables

**Table 1:** Physicochemical and microbial characterization of fermented and non-fermented beverages.

	Non-Fermented Beverage	Fermented Beverage	
	Control	PMO 08	Yogurt
**°Brix (%)**	4.66 ± 0.02 ^NS^	4.63 ± 0.03	4.36 ± 0.01
**Titratable acidity (%)**	0.312 ± 0.016 ^a^	0.958 ± 0.002 ^c^	0.867 ± 0.003 ^b^
**pH**	4.47 ± 0.01 ^a^	3.39 ± 0.03 ^c^	3.60 ± 0.03 ^b^
***Escherichia coli*/coliforms(Log_10_CFU/mL)**	ND	ND	ND
**Yeast and molds(Log_10_CFU/mL)**	ND	ND	ND
**Total aerobic bacteria(Log_10_CFU/mL)**	ND	-	-
**Lactic acid bacteria (Log_10_CFU/mL)**	-	9.78 ± 0.14 *	7.64 ± 0.27

Data are presented as mean ± standard deviation (n = 3). ^a–c^ Values with different superscripts within the same row are significantly different according to Duncan’s multiple range test (*p* < 0.05). * *p* < 0.05, compared with the yogurt starter culture (yogurt) according to the *t*-test. NS, not significantly different. -, not analyzed. ND, not detectable (<1 CFU/mL).

**Table 2:** Changes in the free amino acid composition of fermented beverages.

Amino Acid (mg/100 g)	Non-Fermented Beverage			Fermented Beverage					
	Control			PMO 08			Yogurt		
**Threonine**	6.22	±	0.005 ^a^	4.30	±	0.065 ^c^	5.50	±	0.050 ^b^
**Tyrosine**	2.66	±	0.001 ^a^	0.94	±	0.025 ^c^	2.21	±	0.015 ^b^
**Arginine**	2.44	±	0.010 ^a^	1.16	±	0.025 ^c^	1.76	±	0.010 ^b^
**Alanine**	14.63	±	0.005 ^a^	8.58	±	0.110 ^c^	10.91	±	0.080 ^b^
**Proline**	4.36	±	0.040 ^c^	4.82	±	0.210 ^a^	4.71	±	0.285 ^ab^
**Lysine**	2.58	±	0.030 ^a^	1.52	±	0.190 ^b^	1.26	±	0.155 ^b^
**Histidine**	3.87	±	0.045 ^a^	3.26	±	0.055 ^c^	3.55	±	0.015 ^b^
**Isoleucine**	2.12	±	0.010 ^a^	0.00	±	0.000 ^c^	0.67	±	0.045 ^b^
**Leucine**	2.37	±	0.135 ^a^	0.25	±	0.000 ^c^	1.16	±	0.035 ^b^
**Methionine**	0.74	±	0.050 ^a^	0.00	±	0.000 ^c^	0.00	±	0.000 ^b^
**Phenylalanine**	7.33	±	0.005 ^a^	4.91	±	0.045 ^c^	6.02	±	0.005 ^b^
**Valine**	1.66	±	0.026 ^a^	0.00	±	0.00 ^c^	0.72	±	0.06 ^b^
**Glutamic acid**	126.05	±	0.020 ^a^	109.78	±	1.97 ^c^	107.86	±	1.61 ^b^
**Aspartic acid**	70.00	±	1.780 ^a^	65.05	±	0.27 ^b^	59.55	±	2.14 ^c^
**Serine**	6.48	±	0.010 ^a^	2.86	±	0.06 ^c^	5.81	±	0.02 ^b^
**Glycine**	1.35	±	0.005 ^a^	0.75	±	0.02 ^c^	0.77	±	0.07 ^b^
**Glutamine**	0.21	±	0.005 ^c^	0.83	±	0.00 ^b^	3.79	±	0.39 ^a^

All values are presented as mean ± standard deviation (n = 3). Control: Non-fermented tomato juice beverage; PMO 08: Tomato juice fermented by *L. plantarum* PMO 08; Yogurt: Tomato juice fermented by yogurt starter culture containing *Lactobacillus acidophilus, Streptococcus thermophilus*, and *Bifidobacterium lactis*. ^a–c^ Values with different superscripts within the same row are significantly different according to Duncan’s multiple range test (*p* < 0.05).
